# Comparative Study of Speckle Tracking Echocardiography in Normal and Hypertrophic Cardiomyopathy Cats

**DOI:** 10.3390/vetsci13030277

**Published:** 2026-03-17

**Authors:** Cho-Rok Jeong, Yoon-Joo Shin, Chul Park

**Affiliations:** 1 24H Yedam Animal Medical Center 122, Yeonseo-ro, Eunpyeong-gu, Seoul 03399, Republic of Korea; chorouk@jbnu.ac.kr; 2 Department of Veterinary Internal Medicine, College of Veterinary Medicine, Jeonbuk National University, Iksan 54596, Republic of Korea; yoon416@jbnu.ac.kr

**Keywords:** echocardiography, feline cardiology, feline hypertrophic cardiomyopathy, speckle tracking echocardiography

## Abstract

**Background**: Speckle tracking echocardiography (STE) is a well-established tool in human cardiology for detecting subtle myocardial dysfunction using strain indices. In cats with hypertrophic cardiomyopathy (HCM), STE has been applied in several studies and has identified myocardial deformation abnormalities. This study aimed to identify sensitive echocardiographic markers of myocardial dysfunction in cats with HCM by comparing global strain and strain rate parameters with those of healthy cats. **Methods**: Sixty cats were examined, including 31 healthy controls and 29 HCM-affected cats. Echocardiographic assessments included global circumferential strain (GCS), global radial strain (GRS), global longitudinal strain (GLS), their corresponding strain rates (GCSR, GRSR, and GLSR), left atrial ejection fraction (LAEF), and atrial reservoir strain (RS). **Results**: GLS and GRS were significantly lower in HCM cats than in controls, while GCS showed no significant difference. Among strain rate parameters, only GRSR was significantly reduced in the HCM group. Additionally, both LAEF and RS were markedly decreased, suggesting atrial dysfunction associated with HCM. **Conclusions**: These findings indicate that GLS and GRS are reliable indicators of left ventricular dysfunction in feline HCM and that GRSR may offer additional insight into myocardial deformation dynamics. Overall, STE provides a useful, non-invasive tool for improving the diagnosis and clinical evaluation of feline HCM.

## Simple Summary

Hypertrophic cardiomyopathy (HCM) is the most common heart disease in cats and can lead to heart failure or sudden death. Detecting heart problems early is important, but it can be difficult using standard ultrasound methods. This study used a special imaging technique called speckle tracking echocardiography (STE), which measures how the heart muscle moves and stretches during each heartbeat. We compared heart movement patterns between healthy cats and cats with HCM. The results showed that cats with HCM had weaker heart muscle motion, especially in the directions of length and thickness, and their heart’s pumping and relaxation functions were reduced. These findings suggest that this imaging method can help veterinarians detect early or subtle heart problems that might not be visible with regular scans. By improving early diagnosis and treatment, this research may contribute to better heart health and longer lives for cats affected by heart disease.

## 1. Introduction

HCM is the most common cardiac disease in cats, which is characterized by abnormal thickening of the left ventricular myocardium without other systemic or cardiovascular conditions [1,2,3,4]. Cats exhibit a wide range of clinical signs with variable severity, from asymptomatic cases to congestive heart failure. Diagnosis is primarily established by echocardiography, which allows assessment of cardiac structure and function [5,6].

Traditional echocardiography, using two-dimensional (2D) imaging, M-mode, and Doppler techniques, is key in diagnosing and managing HCM in cats. Key parameters include interventricular septum thickness, left ventricular posterior wall thickness, left atrial diameter, fractional shortening, ejection fraction, and Doppler imaging. While valuable, traditional echocardiography has limited sensitivity for detecting early myocardial changes and subtle functional impairments, necessitating advanced imaging techniques [7].

STE is an advanced modality that tracks natural acoustic markers within myocardial tissue throughout the cardiac cycle [8,9,10,11]. STE quantifies myocardial deformation, providing detailed strain and strain rate information, which are indicators of myocardial function. STE can identify subclinical myocardial dysfunction even when traditional echocardiographic parameters appear normal [12,13].

In human patients with hypertrophic cardiomyopathy (HCM), speckle tracking echocardiography (STE) is widely used and has provided valuable insights into impaired myocardial mechanics [14]. In veterinary cardiology, STE has also been increasingly applied over the past decade, including in cats with HCM, demonstrating its feasibility and clinical relevance [12,15]. Previous feline studies have reported associations between reduced left atrial strain and an increased risk of atrial fibrillation and thromboembolism [14,15], highlighting the potential utility of STE in the assessment of feline HCM. Despite these advances, the clinical application of STE in cats remains relatively limited compared to human cardiology, and further studies are needed to better characterize myocardial deformation patterns and their clinical implications across different stages of feline HCM.

Understanding myocardial function in HCM requires analyzing deformation pat-terns in different dimensions: longitudinal, circumferential, and radial [16]. In healthy hearts, coordinated contraction ensures efficient blood ejection from the left ventricle. HCM disrupts this coordination, altering strain values [5,17].

Although disease presentation is similar, myocardial characteristics differ between humans and cats. The human heart is larger and structurally more complex, operating at a slower heart rate, whereas the feline heart is smaller and functions at a higher heart rate, resulting in distinct hemodynamic stresses. These physiological differences underscore the need for species-specific diagnostic approaches [18].

Evaluating STE’s applicability and accuracy in cats with HCM is crucial, given the anatomical and functional differences between human and feline hearts [12]. Assessing myocardial strain in cats using STE could provide valuable diagnostic and prognostic information, aligning veterinary cardiology practices more closely with human medicine.

This study aims to perform a comparative evaluation of feline patients with HCM using STE and to contrast these findings with those of healthy controls. We hypothesize that HCM patients will exhibit significant alterations in circumferential, radial, and longitudinal strain parameters compared with normal subjects.

## 2. Materials and Methods

### 2.1. Study Population

The study population consisted of 31 client-owned healthy cats and 29 client-owned cats diagnosed with HCM. All cats were adults (>1 year old), weighing 2–6 kg, and included various breeds and both sexes. The HCM group comprised both asymptomatic and stable symptomatic cases. Diagnosis of HCM was established in accordance with the current international consensus guidelines for feline cardiomyopathies [6] and is based on echocardiographic evidence of left ventricular hypertrophy in the absence of other causes. Inclusion criteria required the absence of clinical signs of systemic illness or non-cardiac disease. All cats underwent a complete physical examination, systolic blood pressure measurement, and hematological and biochemical testing, including serum total thyroxine (T4). Cardiac evaluation included echocardiography, electrocardiography, and thoracic radiography, which were all performed without sedation.

Healthy cats were defined as those with an interventricular septal and/or left ventricular free-wall thickness (IVSd and LVFWd) ≤ 6 mm during end-diastole, which were measured by two-dimensional imaging in the left ventricular long-axis and M-mode imaging in the short-axis from the right parasternal view. Additional criteria included systolic blood pressure ≤ 160 mmHg (non-invasive oscillometric measurement), normal serum T4 concentrations, and absence of clinical signs of hyperthyroidism.

Cats were diagnosed with HCM when IVSd and/or LVFWd exceeded 6 mm during end-diastole, which were assessed by the same echocardiographic methods. Cats with congenital heart disease, hyperthyroidism, or systemic hypertension (systolic blood pressure > 160 mmHg by Doppler) were excluded, whereas those receiving cardiac medication were eligible. Healthy controls were confirmed by normal findings on clinical, laboratory, and echocardiographic examinations.

### 2.2. Echocardiographic Acquisition and Analysis

Two-dimensional STE was performed using the left apical four-chamber view to assess longitudinal and left atrial strain and the right parasternal short-axis view at the level of the chordae tendineae for circumferential and radial strain. Images were acquired as cine loops gated to the QRS complex and analyzed offline using dedicated software (EchoPAC PC, GE Medical Systems, Seoul, Korea).

The software automatically tracked myocardial motion and divided each image into six regional segments (anteroseptum, anterior, lateral, posterior, inferior, and septum for circumferential/radial strain; base-septum, middle-septum, apical-septum, apical-lateral, middle-lateral, and base-lateral for longitudinal strain). Tracking quality was validated by the program, and manual adjustment of the endocardial border was performed if necessary. Analyses were based on complete cardiac cycles determined by simultaneous ECG R–R intervals.

### 2.3. STE Analysis

STE was performed to evaluate myocardial strain and strain rate parameters. By tracking the motion of natural acoustic markers (speckles) within the myocardial tissue, STE provides quantitative assessment of myocardial deformation in three principal directions: longitudinal, circumferential, and radial.

Global longitudinal strain (GLS) was calculated as the mean of longitudinal strain values obtained from the endocardial, mid-myocardial, and epicardial layers: GLS = (εendo + εmid + εepi)/3.

**Figure 1:**
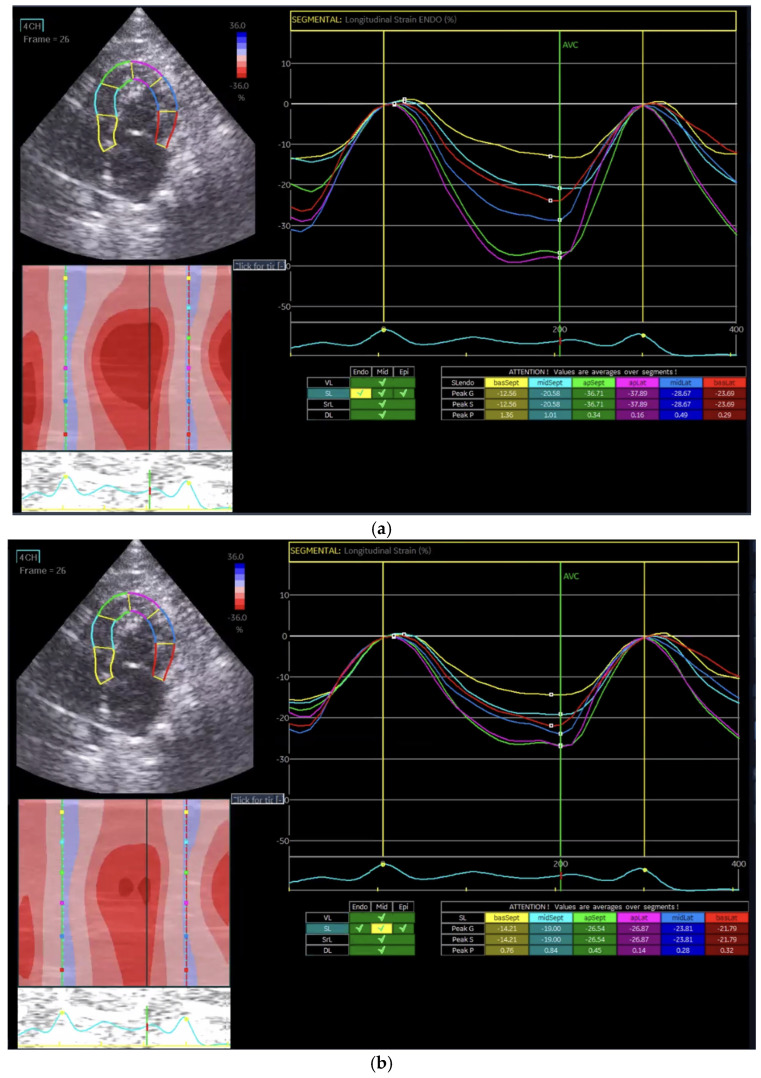

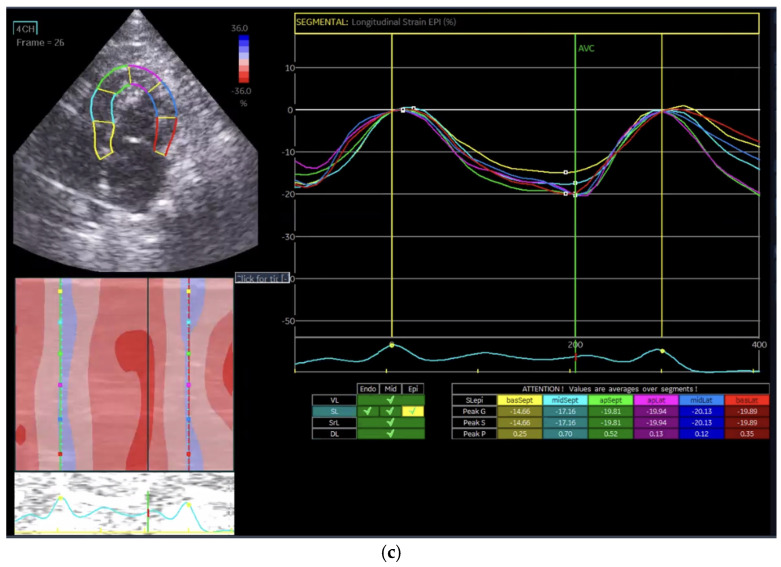
Global longitudinal strain (GLS) analysis in a feline heart using speckle tracking echocardiography (STE). (**a**) Endocardial longitudinal strain analysis; (**b**) mid-myocardial longitudinal strain analysis; (**c**) epicardial longitudinal strain analysis.

Longitudinal strain represents myocardial fiber deformation along the long axis of the heart, which is expressed as a negative percentage during systolic shortening. Endocardial longitudinal strain (Figure 1a) is highly sensitive to ischemic or subclinical myocardial dysfunction, whereas mid-myocardial (Figure 1b) and epicardial strains (Figure 1c) provide additional information on overall and outer-layer function, respectively.

**Figure 2:**
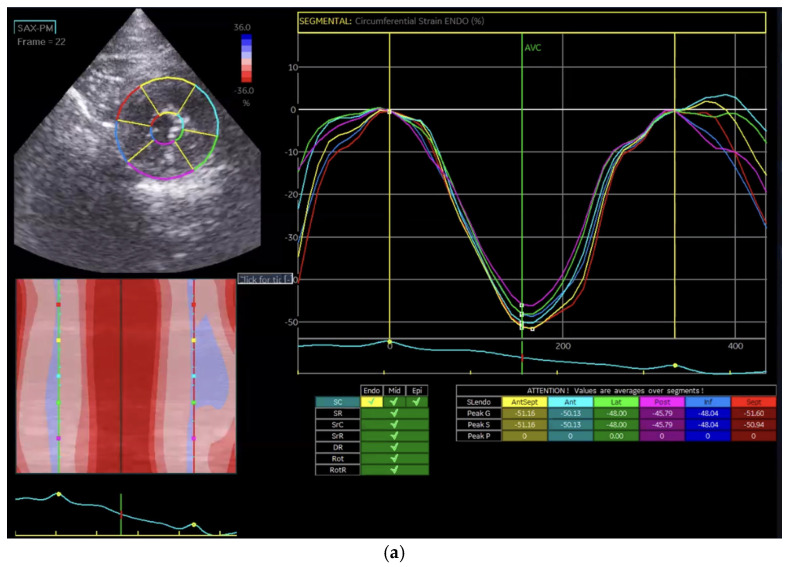

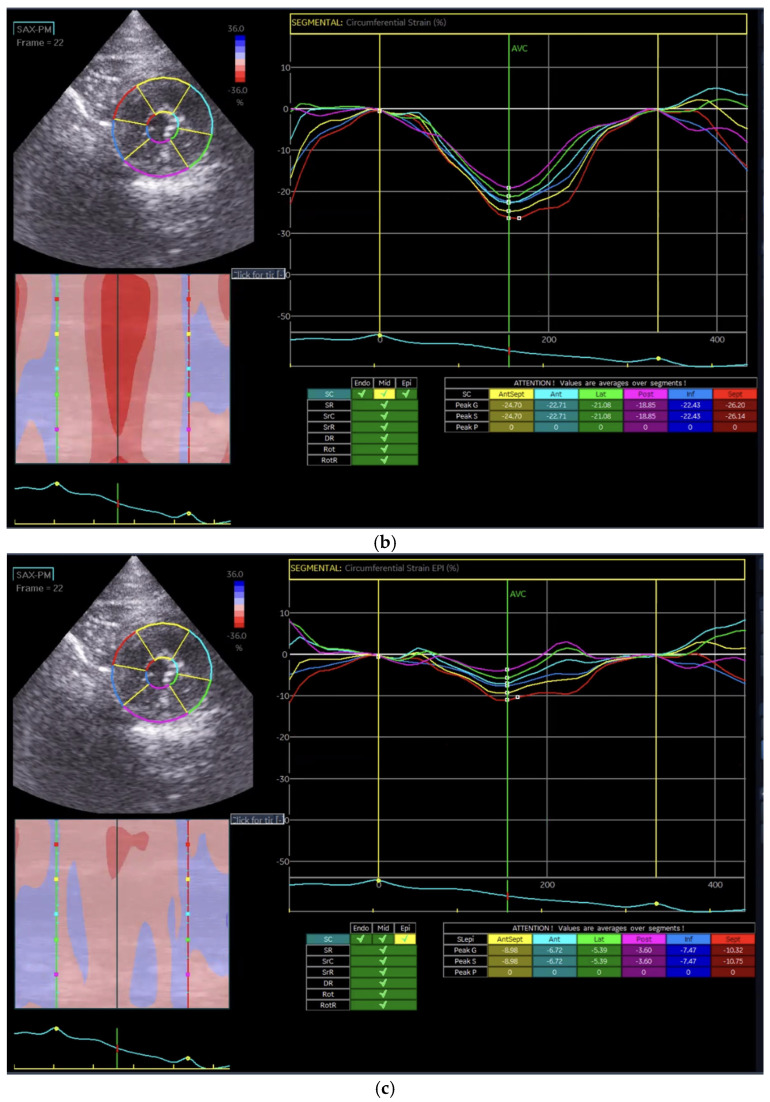
Global circumferential strain (GCS) analysis in a feline heart using STE. (**a**) Endocardial circumferential strain analysis; (**b**) mid-myocardial circumferential strain analysis; (**c**) epicardial circumferential strain analysis.

Global circumferential strain (GCS) was calculated in a similar manner by averaging circumferential strain values across the endocardial (Figure 2a), mid-myocardial (Figure 2b), and epicardial layers (Figure 2c): GCS = (εendo + εmid + εepi)/3.

Circumferential strain evaluates myocardial fiber deformation around the ventricular circumference and is particularly useful for detecting concentric remodeling, such as in hypertrophic cardiomyopathy.

**Figure 3:**
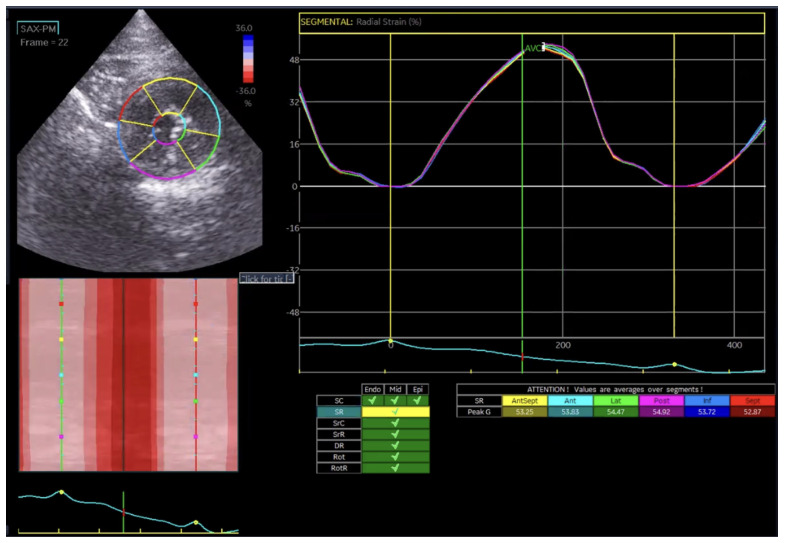
Global radial strain (GRS) analysis in a feline heart using STE.

Global radial strain (GRS) reflects myocardial wall thickening from endocardium to epicardium and was analyzed as a single value, since layer-specific segmentation is not physiologically applicable for radial deformation (Figure 3).

**Figure 4:**
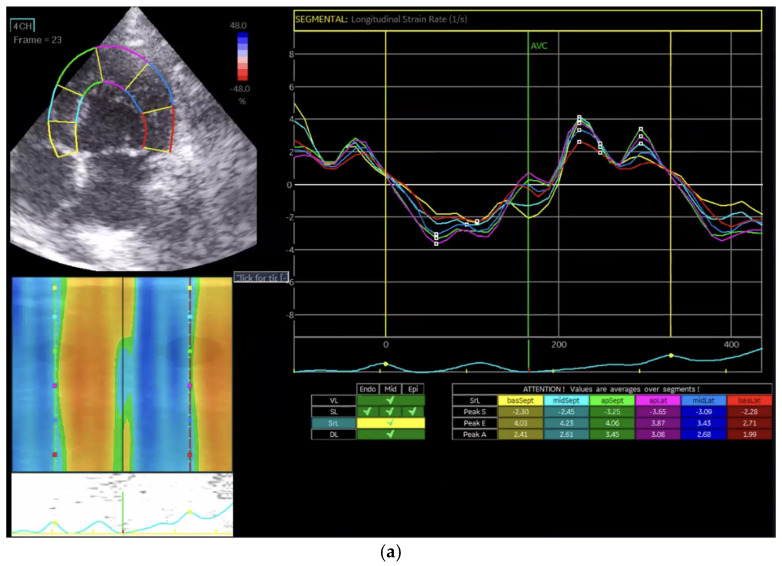

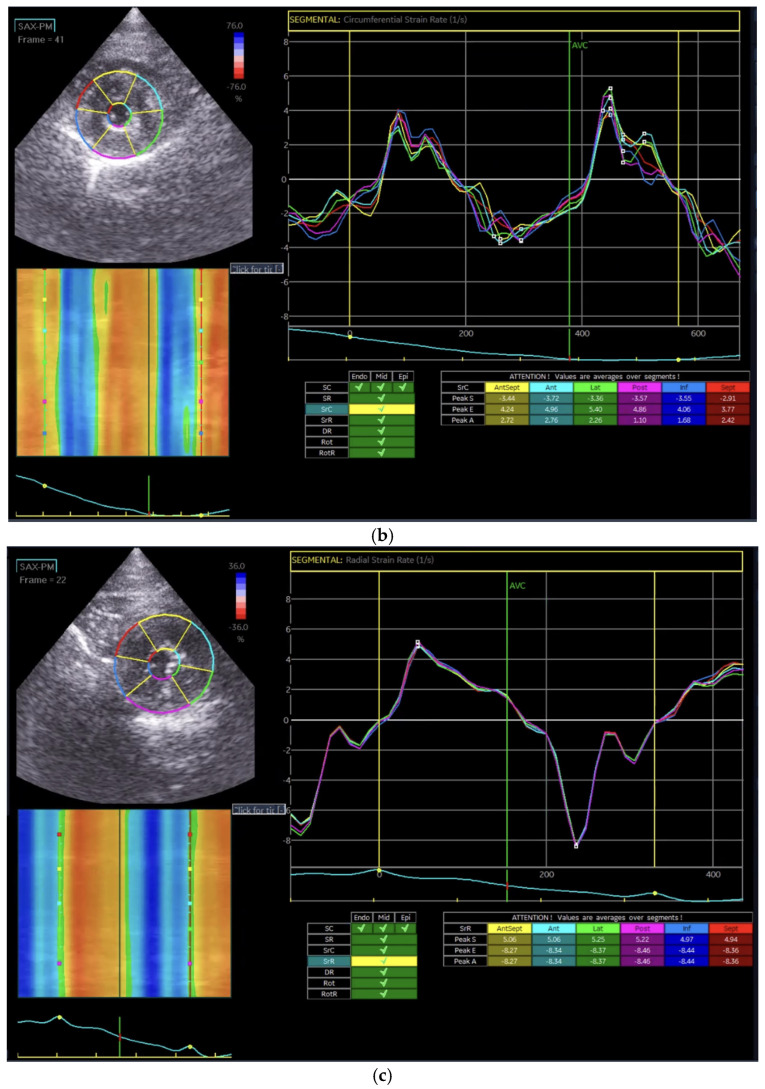
Strain rate analysis in a feline heart using STE. (**a**) Longitudinal strain rate (GLSR) analysis; (**b**) circumferential strain rate (GCSR) analysis; (**c**) radial strain rate (GRSR) analysis.

Strain rate (SR) was calculated as the temporal derivative of strain, representing the rate of myocardial deformation per unit of time. Longitudinal (GLSR, Figure 4a), circumferential (GCSR, Figure 4b), and radial strain rates (GRSR, Figure 4c) were analyzed separately to evaluate contraction and relaxation dynamics in each direction.

**Figure 5:**
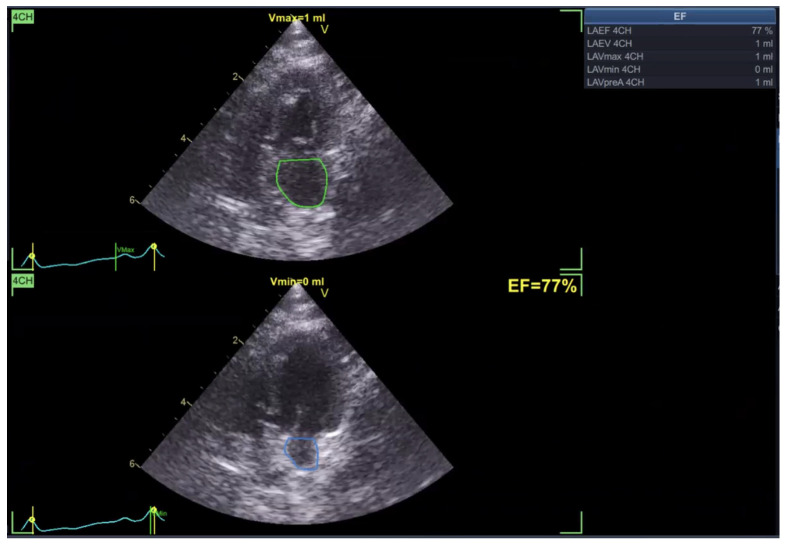
Left atrial ejection fraction (LAEF) analysis in a feline heart using STE.

**Figure 6:**
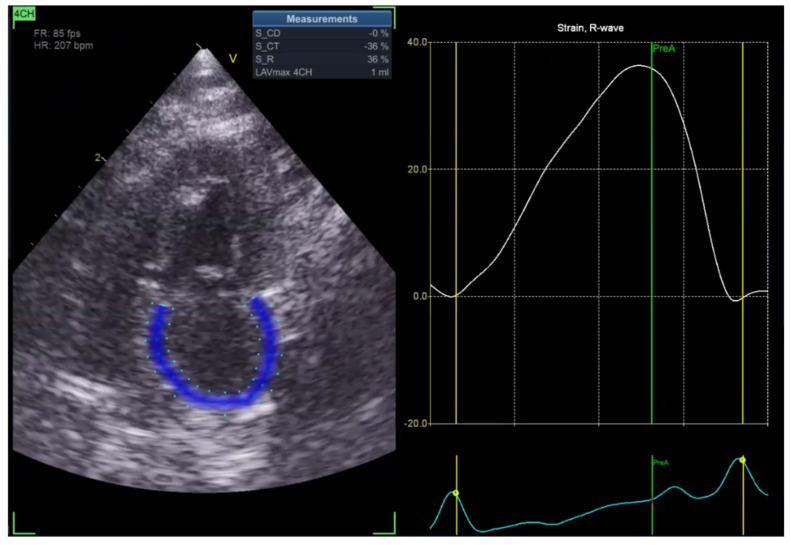
Reservoir strain rate (RS) analysis in a feline heart using STE.

Left atrial ejection fraction (LAEF) was calculated as the percentage of blood volume ejected during atrial contraction (Figure 5). In addition, reservoir strain rate (RS) was measured to quantify atrial deformation during the reservoir phase, which is when the left atrium fills from the pulmonary veins with the mitral valve closed (Figure 6).

### 2.4. Statistical Analysis

Statistical analyses were conducted using IBM SPSS Statistics version 22 (IBM Corp., Armonk, NY, USA). Descriptive statistics (mean ± standard deviation) were calculated for all echocardiographic parameters. Comparisons between Normal and HCM groups were performed using independent-sample *t*-tests, with significance set at *p* < 0.05.

### 2.5. Ethical Approval

This study did not involve experimental procedures and therefore did not require approval from an Institutional Animal Care and Use Committee (IACUC). All examinations were part of routine clinical evaluations, and informed consent was obtained from the owners of all participating cats.

## 3. Results

**Figure 7:**
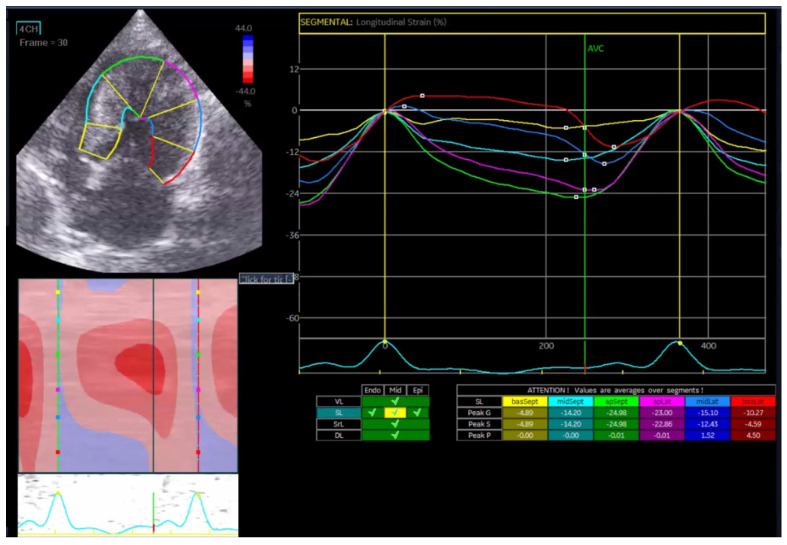
Longitudinal strain (GLS) analysis in a feline HCM patient using speckle tracking echocardiography (STE).

**Figure 8:**
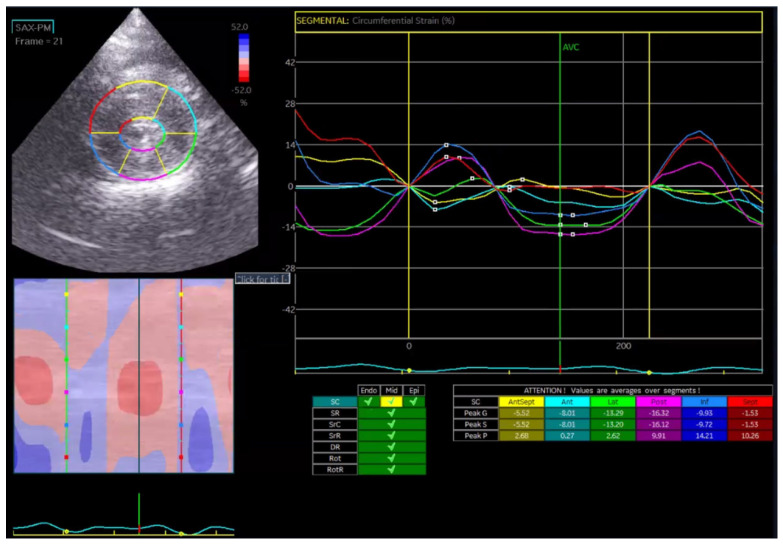
Circumferential strain (GCS) analysis in a feline HCM patient using speckle tracking echocardiography (STE).

**Figure 9:**
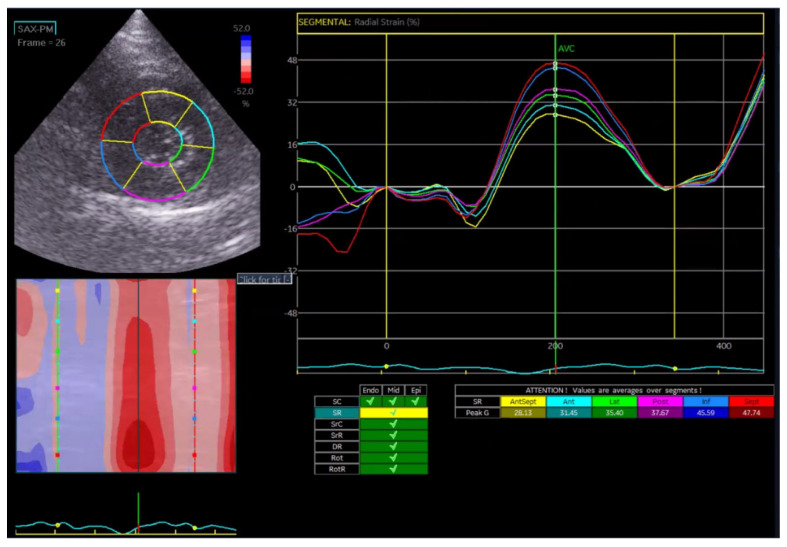
Radial strain (GRS) analysis in a feline HCM patient using speckle tracking echocardiography (STE).

**Figure 10:**
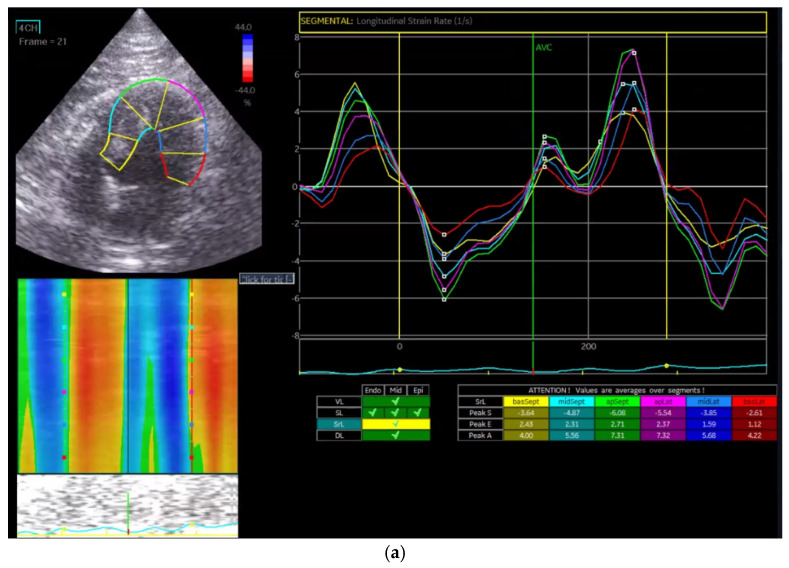

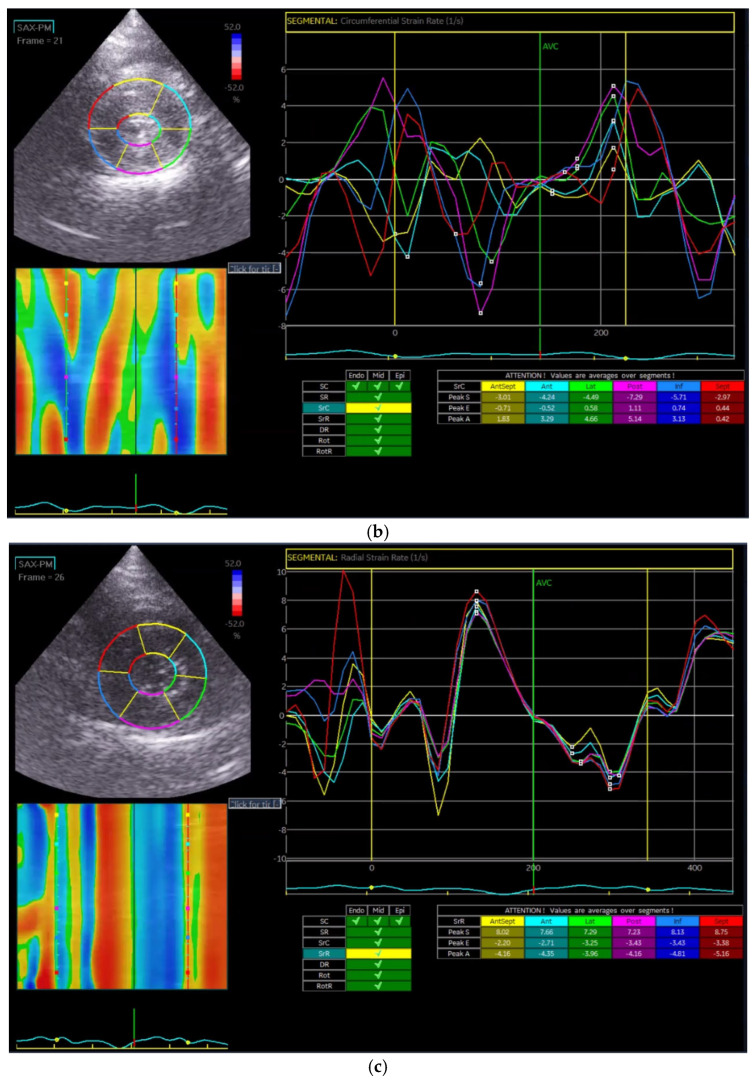
Strain rate analysis in a feline HCM patient using STE. (**a**) Longitudinal strain rate (GLSR) analysis; (**b**) circumferential strain rate (GCSR) analysis; (**c**) radial strain rate (GRSR) analysis.

**Figure 11:**
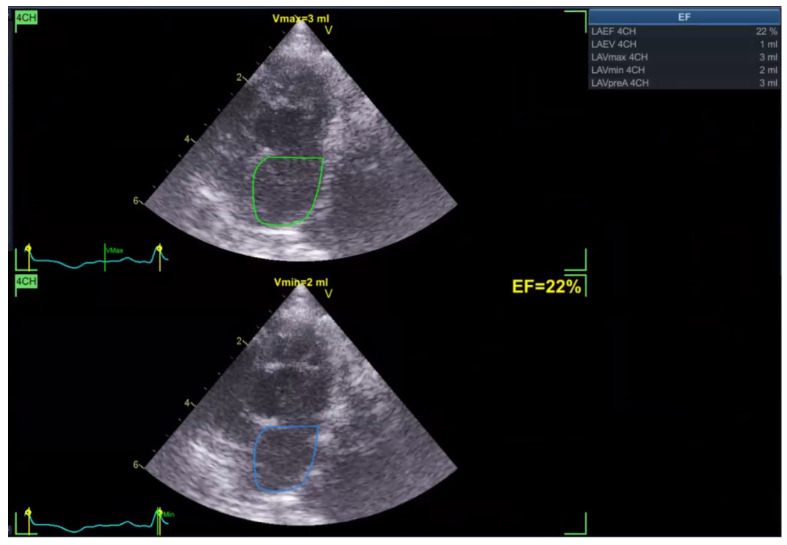
Left atrial ejection fraction (LAEF) analysis in a feline HCM patient using STE.

**Figure 12:**
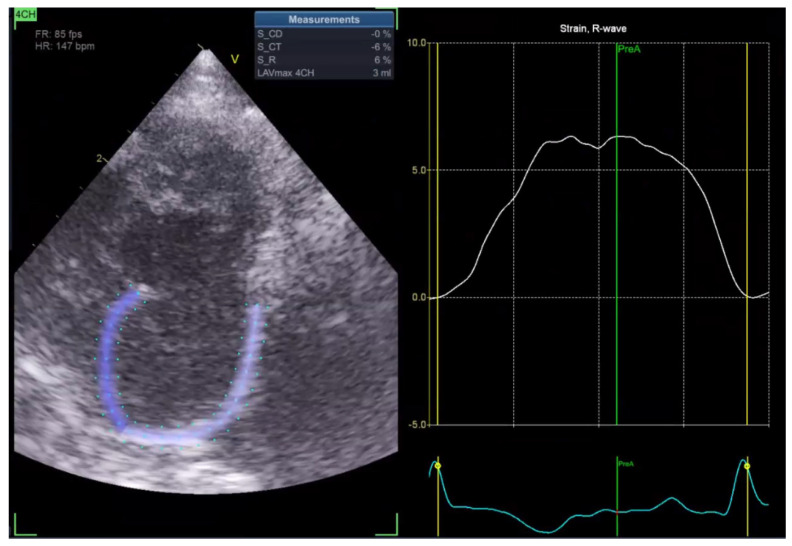
Reservoir strain rate (RS) analysis in a feline HCM patient using STE.

In the Normal group (n = 31), the mean age was 7.39 ± 4.48 years, body weight was 5.04 ± 1.74 kg, heart rate was 182.35 ± 24.40 bpm, and systolic blood pressure was 134.35 ± 9.38 mmHg. In the HCM group (n = 29), the mean age was 6.72 ± 3.95 years, body weight was 5.13 ± 1.59 kg, heart rate was 206.55 ± 29.05 bpm, and systolic blood pressure was 133.79 ± 24.01 mmHg. Age and body weight did not differ significantly between the two groups, whereas heart rate and systolic blood pressure were significantly different (*p* < 0.05). The STE analyses of the Normal cat group are presented in Figure 1, Figure 2, Figure 3, Figure 4, Figure 5 and Figure 6, and those of the cats with HCM are shown in Figure 7, Figure 8, Figure 9, Figure 10, Figure 11 and Figure 12.

The distribution of STE parameters within the HCM group, along with the mean values and standard deviations for both the HCM and Normal groups, are summarized in Table 1. The results of independent *t*-tests comparing the two groups are also presented in the same table, demonstrating variable differences across the measured echocardiographic indices.

As summarized in Table 1, the distribution of STE parameters within the HCM group, together with the mean values and standard deviations for both the HCM and Normal groups, is presented alongside the outcomes of statistical evaluations. Comparative analyses revealed that, among the global strain parameters, GRS was significantly reduced in the HCM group compared with the Normal group (*p* = 0.027). A similar pattern was observed for global longitudinal strain (GLS), which was markedly lower in HCM cats (*p* = 0.0126), whereas no significant difference was detected for GCS (*p* = 0.278). With respect to strain rate indices, the GRSR was significantly decreased in the HCM group (*p* = 0.046), while the GCSR (*p* = 0.633) and GLSR (*p* = 0.452) did not differ significantly between groups. In addition, LAEF was significantly diminished in HCM cats (*p* = 0.0097), and RS was also markedly reduced compared with controls (*p* = 0.0058).

Taken together, the statistical analyses demonstrated significant group differences in GRS, GLS, GRSR, LAEF, and RS, whereas GCS, GCSR, and GLSR did not show significant variation between the groups.

## 4. Discussion

In human medicine, GCS, GRS, and GLS are established parameters that exhibit significant differences between healthy individuals and patients with HCM [19]. These strain parameters are typically reduced in HCM patients, reflecting myocardial deformation abnormalities and fibrosis characteristics of the disease [20]. Consequently, strain measurements are considered valuable diagnostic and prognostic markers, offering insights into myocardial mechanics beyond conventional indices such as ejection fraction [1,13].

Research on STE in cats, particularly in the context of HCM, remains limited [12]. Nevertheless, previous studies have reported significant differences in GCS, GRS, and GLS between normal cats and those with HCM [21,22], suggesting that these parameters could similarly aid in the diagnosis and monitoring of feline HCM [23]. Early detection of HCM through strain analysis may enable timely intervention and improve clinical outcomes.

In contrast to earlier studies, the present investigation did not identify significant differences in GCS between normal and HCM cats [4]. However, both GRS and GLS were significantly reduced in HCM cats, which is consistent with myocardial dysfunction due to hypertrophy, fibrosis, and myofiber disarray. These findings indicate that GRS and GLS may serve as sensitive indicators of myocardial dysfunction in feline HCM. The absence of significant differences in GCS aligns with some previous reports [12,24], suggesting that circumferential fibers may be less consistently affected in cats or that current methodologies may have limited sensitivity in detecting subtle changes. Further investigation is warranted to clarify the role of GCS in feline HCM.

In addition, a significant difference in GRSR was observed between groups, whereas GCSR and GLSR were not significantly different. This finding suggests that the rate of radial deformation is particularly affected in feline HCM and may represent a more sensitive parameter for detecting early or subtle myocardial changes [25,26]. Previous studies have suggested that strain parameters may be more sensitive to early myocardial dysfunction than strain rate indices, as deformation can be impaired before measurable changes in the rate of deformation occur [27,28]. In cats with hypertrophic cardiomyopathy, radial strain has been reported to be particularly sensitive to myocardial dysfunction, supporting the potential clinical value of GRS and GRSR in disease detection [29]. Interestingly, while GLS differed significantly between groups, GLSR did not, implying that myocardial deformation may be impaired in the early stages of disease progression, whereas the rate of deformation may only be altered at more advanced stages. This observation underscores the potential of myocardial strain, rather than strain rate, as an early marker of dysfunction.

Heart rate was significantly higher in cats with HCM, which may have influenced myocardial strain measurements by reducing diastolic filling time and altering ventricular relaxation. Previous studies have shown that increased heart rate can affect myocardial deformation and strain measurements [30], as well as diastolic filling dynamics and indices of diastolic function, including E/E′ and intraventricular pressure gradients [31].

Nevertheless, the clinical application of STE should be interpreted in light of its technical and methodological limitations, including dependence on image quality, frame rate, heart rate, and vendor-specific analysis algorithms [32,33]. These factors may influence measurement variability, particularly in cats with small cardiac dimensions and high heart rates. Therefore, STE-derived parameters should be considered complementary to, rather than replacements for, conventional echocardiographic indices.

Within these limitations, STE holds promise as an adjunctive tool for the early detection and monitoring of myocardial dysfunction in feline HCM, particularly in cases with preserved systolic function on conventional echocardiography. One limitation of this study is the relatively small sample size, which may not fully capture the heterogeneity of disease progression in feline hypertrophic cardiomyopathy; therefore, future studies including larger cohorts are warranted. Overall, this study contributes to the growing body of evidence supporting the role of advanced echocardiographic techniques in improving the diagnosis and clinical assessment of hypertrophic cardiomyopathy in feline patients. In future studies, we plan to investigate longitudinal changes in diagnostic patterns according to disease progression and to evaluate their associations with related complications through continuous follow-up monitoring.

## 5. Conclusions

This study demonstrates that STE provides valuable insights into myocardial function in cats with HCM. Among the parameters evaluated, GRS and GLS were significantly reduced in HCM cats, indicating their reliability as sensitive markers of myocardial dysfunction. Additionally, the decrease in GRSR, LAEF, and atrial RS suggests that both ventricular and atrial functions are affected by the disease. In contrast, the absence of significant differences in GCS highlights the variability in circumferential fiber involvement and the potential limitations of current methodologies. Overall, these findings support the clinical utility of STE as a non-invasive diagnostic tool for detecting and monitoring myocardial and atrial dysfunction in feline HCM. Further research is warranted to refine measurement techniques and establish standardized reference values to enhance its application in veterinary cardiology.

## Figures and Tables

**Table 1:** Descriptive statistics (mean ± SD) and statistical results comparing measured variables between Normal and HCM groups.

Variable	Normal (Mean ± SD)	HCM (Mean ± SD)
GLS	−23.63 ± 5.63	−18.79 ± 7.62 ^1^
GCS	−25.98 ± 7.25	−24.11 ± 12.87
GRS	47.73 ± 21.22	37.89 ± 20.15 ^1^
GLSR	−3.279 ± 0.988	−3.066 ± 1.114
GCSR	−4.174 ± 2.001	−4.464 ± 2.259
GRSR	6.171 ± 1.545	4.951 ± 1.995 ^1^
LAEF	59.24 ± 9.73	49.36 ± 16.92 ^2^
RS	21.86 ± 9.54	14.93 ± 8.67 ^2^

^1^ Statistically significant difference compared to normal (*p* < 0.05). ^2^ Statistically significant difference compared to normal (*p* < 0.01).

## Data Availability

The data presented in this study are available on request from the corresponding author due to ethical and privacy restrictions related to clinical patient data, and any access will require institutional and ethical approvals.

## Abbreviations

The following abbreviations are used in this manuscript:

STE	Speckle Tracking Echocardiography
GCS	Global Circumferential Strain
GRS	Global Radial Strain
GLS	Global Longitudinal Strain
HCM	Hypertrophic Cardiomyopathy
LAEF	Left Atrial Ejection Fraction
RS	Reservoir Strain Rate
IVSd	Interventricular Septal Thickness
IVFWd	Left Ventricular Free-Wall Thickness
IACUC	Institutional Animal Care and Use Committee
GRSR	Global Radial Strain Rate
GCSR	Global Circumferential Strain Rate
GLSR	Global Longitudinal Strain Rate
